# A Case of Depersonalization-Derealization Disorder

**DOI:** 10.1192/bjo.2025.10752

**Published:** 2025-06-20

**Authors:** Emeka Onochie, Joseph Verghese

**Affiliations:** 1 Nottinghamshire Healthcare NHS Foundation Trust, Nottingham, United Kingdom; 2 lincolnshire Partnership NHS Foundation Trust, Stamford, United Kingdom

## Abstract

**Aims:** Background: Depersonalization-derealization disorder (DPDR), classified under ICD–11 code 6B66, involves persistent or recurrent experiences of depersonalization, derealization, or both. Depersonalization refers to a sense of detachment from one’s thoughts, emotions, or body, whereas derealization involves perceiving the external world as unreal or distorted. These symptoms cause significant distress or impairment, are not attributable to other mental disorders, substance use, or medical conditions, and occur while reality testing remains intact.

**Methods:** Case Report.

An 18-year-old female A-level student presented with a two-year history of frequent episodes in which her surroundings, including people and familiar environments, felt unreal. These episodes varied in duration and were highly distressing, particularly during emotional extremes such as heightened happiness or stress. Symptoms were most pronounced in the evenings or when she was unoccupied, leading to emotional breakdowns. Despite these experiences, she remained aware of her own reality, with disturbances centred on external perceptions.

Her symptoms began following a nine-month psychiatric hospitalization. Prior to admission, she experienced unexplained gastrointestinal symptoms, and in the absence of an identifiable physical cause, she was diagnosed with conversion disorder. The hospitalization was distressing due to frequent invasive procedures, a perceived sense of blame for her condition, and feelings of entrapment. She subsequently developed post-traumatic stress disorder (PTSD), characterized by flashbacks, nightmares, and avoidance of medical settings. However, DPDR symptoms persisted outside of PTSD-related re-experiencing episodes, causing ongoing distress and impairment.

**Results:** Discussion: This case highlights the complex interplay between DPDR and PTSD, particularly following medical trauma. While dissociative symptoms frequently occur in PTSD, ICD–11 differentiates DPDR as a distinct disorder when symptoms persist beyond re-experiencing episodes. In this case, the patient’s prolonged hospitalization, combined with perceived invalidation and invasive interventions, likely contributed to the development of DPDR as a maladaptive dissociative response.

The exacerbation of symptoms during emotional extremes aligns with research indicating that dissociation may function as an affect regulation mechanism. Trauma-related dissociation has been linked to disruptions in emotional processing, potentially interfering with adaptive coping strategies. This underscores the importance of targeted psychological interventions to reduce distress and improve functional outcomes.

**Conclusion:** A trauma-informed, multidisciplinary approach is essential in managing this patient’s complex presentation. Psychological interventions such as EMDR or trauma-focused CBT should be integrated with ongoing medical care to address both dissociative symptoms and physical health concerns. Collaborative management between psychiatric and medical teams will be crucial in promoting long-term recovery, enhancing her psychological resilience, and improving overall quality of life.

